# The psychological burden associated with metabolic syndrome: Evidence from UK and US older adults

**DOI:** 10.1002/osp4.780

**Published:** 2024-07-06

**Authors:** Eric Robinson, Michael Daly, I Gusti Ngurah Edi Putra

**Affiliations:** ^1^ Department of Psychology Institute of Population Health University of Liverpool Liverpool UK; ^2^ Department of Psychology Maynooth University Maynooth Ireland

**Keywords:** metabolic syndrome, non‐communicable diseases (NCDs), psychological well‐being

## Abstract

**Introduction:**

We examined the psychological burden associated with metabolic syndrome (MetSyn).

**Methods:**

We used comparable longitudinal data of older adults (≥50 years) from the UK (English Longitudinal Study of Aging) and the US (Health and Retirement Study). We defined MetSyn based on biomarker assessments (e.g., blood pressure, impaired glycemic control). Using regression models, we tested a range of individual psychological outcomes (e.g., depressive symptoms) associated with MetSyn. We also examined whether these psychological outcomes may explain or moderate the link between MetSyn and non‐communicable diseases (NCDs).

**Findings:**

MetSyn was associated cross‐sectionally with a range of psychological outcomes, including depression, anxiety, loneliness, hopelessness, cynical hostility, social strain, negative affect and decreased positive affect, social support and purpose in life. There was no convincing evidence that psychological factors interacted with or explained (mediated) the relationship between MetSyn and increased risk of developing NCD over 10‐year follow‐ups.

**Conclusions:**

MetSyn and the psychological burden outcomes examined may have independent effects on NCD risk.

## 1 INTRODUCTION

Metabolic syndrome (MetSyn) is a well establish risk factor for development of non‐communicable diseases (NCDs), such as heart disease and stroke.^1^, ^2^ Conceptually related to MetSyn is obesity and there is a sizable psychosocial burden associated with obesity that is thought to detrimentally impact the management of obesity and obesity‐related health outcomes.^3^ Like obesity, MetSyn has also been shown to be associated with worse mental health, including increased depression and anxiety symptoms.^4^, ^5^ However, the association MetSyn has with a wider range of psychological factors that can shape physical health outcomes is unclear.^6^ In particular, there is limited research examining how MetSyn relates to other markers of mental health (e.g., experiences of positive affect) and psychosocial factors such as social support or loneliness, in the general population.^7^ Moreover, it is important to understand if MetSyn is associated with worse mental health and psychosocial factors, independent of obesity status, as MetSyn has become relatively common in adults in the normal weight and overweight BMI range.^8^ Therefore, in the present research, we examined the association that MetSyn has with a wide range of health‐relevant psychosocial factors, independent to obesity status, among samples of nationally representative UK and US older adults.

Until now, it has been widely assumed that MetSyn increases the risk of ill physical health due to the direct physiological impact MetSyn has on the body (e.g., dysregulation of metabolic functioning, inflammation).^1^ Yet, the psychological burden associated with MetSyn may be important as it could interact with MetSyn (moderation), with those experiencing negative psychological effects of MetSyn being at amplified risk of NCD development. For example, a study of employed US adults found that worse mental health (stress and anxiety) among male participants with MetSyn was associated with increased blood pressure, but no such relationship was observed among participants without MetSyn.^9^ These findings are consistent with the proposition that worse mental health may amplify the damaging effects that MetSyn has on physical health, but there has been limited additional testing of this proposition in the context of disease development.

Alternatively, it is possible that the psychological burden associated with MetSyn may in part explain (mediation) how MetSyn increases the risk of NCD development, as negative psychological factors have been shown to contribute to poorer physiological health. Individuals living with MetSyn experience worse psychological well‐being,^10^ and impairments in psychological well‐being are risk factors for NCD development.^11^, ^12^ Therefore, a currently untested proposition is that impaired psychological well‐being may be partially responsible for why NCD development is more common among those living with MetSyn. Although we are aware of no research directly testing this possibility using longitudinal cohort data, there is a significant body of related research which proposes that the psychological burden of obesity may explain why obesity is associated with worse physical health.^13^

A better conceptualization of the psychological burden associated with MetSyn and whether this burden may contribute to adverse physical health outcomes among those living with MetSyn may provide insights for MetSyn management and treatment provision.^14^ Therefore, the present research examined whether psychological factors have any moderating or mediating MetSyn‐related NCD development risk in later life, as opposed to an independent (additive) effect.

## 2 METHODS

### 2.1 English Longitudinal Study of Aging (ELSA)

English Longitudinal Study of Aging (ELSA) is a representative longitudinal study of older English adults (≥50 years).^15^ Wave 4 (2008–2009) acted as the cross‐sectional baseline in this study due to the range of psychological measures available. Development of NCDs was examined from baseline up to Wave 9 (2018–2019), including biennial measurement at Waves 5 (2010–2011), 6 (2012–2013), 7 (2014–2015), and 8 (2016–2017).

### 2.2 Health and Retirement Study (HRS)

Health and Retirement Study (HRS) is a comparable study of older US adults.^16^ For the present study, data were combined from Waves 9 (2008) and 10 (2010) as baseline. Development of NCDs was examined from baseline up to Wave 15 (2020), including biennial measurements at Waves 11 (2012), 12 (2014), 13 (2016), and 14 (2018).

### 2.3 Metabolic syndrome (MetSyn)

Consistent with previous research,^17^ participants were classed as probable MetSyn if they had ≥2 of the following metabolic risk factors: (1) high‐normal blood pressure (>130/85 mmHg, or self‐reported diagnosed with hypertension), (2) impaired glycemic control (HbA1c ≥6.0% or 42 mmol/mol), (3) low high‐density lipoprotein (HDL)‐cholesterol levels (<1.03 mmol/L in men and <1.30 mmol/L in women), (4) high triglyceride levels (≥1.7 mmol/L), and (5) systemic inflammation (CRP≥3 mg/L). In HRS, triglyceride levels were unavailable. The other four metabolic risk factors were used to define MetSyn status.

### 2.4 Psychological measures

Both ELSA and HRS included validated measures of depressive symptoms, life satisfaction, loneliness, social support and social strain. Eudemonic well‐being and enjoyment of life were measured in ELSA only, and positive and negative affects, purpose in life, anxiety, hopelessness, optimism, pessimism, cynical hostility, personal constraint and mastery were assessed in the HRS only. See Supporting Information S1 for detailed measurement information. We followed our previous approach in quantifying these psychological outcomes.^18^ All measures that were on a continuous scale were standardized using the z‐score method to enable comparison across different metrics.

### 2.5 Non‐communicable disease (NCDs)

In both studies, the risk of participants developing heart disease, stroke, arthritis, cancer and cognitive decline (memory‐related disease) was examined. Diabetes or hypertension were not examined as MetSyn classification was dependent on diagnostic factors. Health conditions were measured via self‐reported doctor diagnosis (“Has a doctor ever told you that you have…?”), with the exception of cognitive decline that was also measured via a telephone interview test for cognitive status (as in^18^). See Supporting Information S1 for full details.

### 2.6 Covariates

Sociodemographic covariates controlled in all analyses were: age (years), sex (male, female), employed (yes, no), ethnicity (White, non‐White), BMI category status (BMI [kg/m^2^: 18.5–24.9, 25–29.9, ≥30, participants with BMI < 18.5 (underweight) excluded from analyses], household wealth (quintiles), marital status and education (degree‐level qualification: yes, no in ELSA versus years in education for HRS).

### 2.7 Analyses

Linear regressions tested the association between MetSyn and each psychological measure at baseline, controlling for covariates (see above). Associations were also examined with a composite measure of psychological measures that loaded onto single factors relating to “Psychological Distress” (see Table 1, five measures in ELSA, 10 measures in HRS). Cox‐proportional hazard regression models examined how MetSyn at baseline related to new diagnosis for each NCD (covariates included). Time to diagnosis was calculated from the date of the interview at baseline to the diagnosis date/follow‐up interview when the NCD was first reported. Participants with the NCDs of interest at baseline were excluded per model. Interaction terms were included between MetSyn status and each psychological measure to test evidence for moderation. Causal mediation analysis was used to examine the role of any psychological measures in explaining the association between MetSyn and NCDs. Inverse probability weighting accounted for loss to follow‐up (see Supporting Information S1 for full information). To account for the large number of analyses conducted, alpha was reduced to <0.01 for mediation and moderation analyses. The analyses were pre‐registered at https://doi.org/10.17605/OSF.IO/JRQAP, with the exception of moderation analyses (unplanned additional analysis).

**TABLE 1:** Cross‐sectional associations between metabolic syndrome status and psychological measures.

	ELSA		HRS	
	*β*	95% CI	*β*	95% CI
Depressive symptoms	0.08	0.02, 0.14**	0.08	0.04, 0.13**
Enjoyment of life	−0.18	−0.24, −0.12***		
Eudemonic well‐being	−0.12	−0.18, −0.06***		
Life satisfaction	−0.04	−0.10, 0.02	−0.07	−0.12, −0.02**
Loneliness	0.06	0.01, 0.12*	0.06	0.01, 0.11*
Social support	−0.07	−0.13, −0.01*	−0.05	−0.10, −0.01*
Social strain	0.07	0.00, 0.13*	0.08	0.03, 0.12**
Positive affect			−0.07	−0.12, −0.02**
Negative affect			0.06	0.01, 0.11*
Purpose in life			−0.08	−0.13, −0.03**
Anxiety			0.10	0.05, 0.15***
Hopelessness			0.07	0.02, 0.12**
Optimism			0.01	−0.04, 0.06
Pessimism			0.08	0.03, 0.13**
Cynical hostility			0.08	0.03, 0.13**
Personal constraint			0.08	0.03, 0.13**
Mastery			−0.01	−0.06, 0.04
Index of psychological distress	0.12	0.06, 0.18***	0.11	0.06, 0.16***

*Note*: Positive *β* indicates presence of metabolic syndrome = higher score for psychological measure. ELSA model sample sizes range from *N* = 5223 to *N* = 5721. HRS model sample sizes range from *N* = 9532 to *N* = 9868. The association between metabolic syndrome and psychological outcomes was examined in separate regression models for each outcome and controlled for age, sex, ethnicity, marital status, employment status, education (degree qualification in ELSA, the number of years in education in HRS), household wealth, and BMI category. Using exploratory factor analysis, psychological measures loaded onto the same underlying single factor, defined as having an item loading factor ≥0.55 (good item loading), were combined into a composite index of psychological distress separately in each study (as in ^18^). Index of psychological distress was developed by re‐standardizing the average standardized scores of 5 psychological measures in ELSA (depressive symptoms, eudemonic wellbeing, enjoyment of life, life satisfaction, loneliness) and 10 psychological measures in HRS (depressive symptoms, life satisfaction, loneliness, positive affect, negative affect, purpose in life, anxiety, hopelessness, pessimism, and personal constraint) that were found to load onto a single factor in factor analysis.

Abbreviations: *β*, regression coefficient; CI, confidence interval.

*p < 0.05; **p < 0.01; ***p < 0.001.

## 3 RESULTS

ELSA (54% female, 66 mean years old) and HRS (55% female, 66 mean years old) were comparable for basic demographics. MetSyn was common in the UK (60%) and US (52%) at baseline. See online Supporting Information S1 for detailed participant characteristics. As indicated in Table 1, MetSyn (vs. its absence) was cross ‐ sectionally associated with a wide range of negative psychosocial outcomes, including increased depression, anxiety, loneliness, hopelessness, cynical hostility, social strain, negative affect and decreased positive affect, social support and purpose in life. In HRS, MetSyn was significantly associated with reduced life satisfaction (but not in ELSA). MetSyn (vs. its absence) was associated with significantly raised levels of psychological distress on the overall composite index in both ELSA (*β* = 0.12, 95% CI: 0.06–0.18) and HRS (*β* = 0.11, 95% CI: 0.06–0.016).

MetSyn status at baseline was associated with increased risk of developing stroke in both ELSA and HRS (hazard ratios ranging from 1.45 to 1.57), heart disease in HRS (HR = 1.32), arthritis in ELSA (HR = 1.21), but not cancer or cognitive decline in both studies (hazard ratios ranging from 0.19 to 1.05). See Tables 2 and 3. There was no evidence that any psychological measures (independently or when combined in composite) significantly moderated or mediated the associations between MetSyn status at baseline and NCD development. See Tables 4 and 5 for moderation analyses in full and online supplementary materials for mediation results.

**TABLE 2:** Longitudinal associations between metabolic syndrome status and the development of non‐communicable health conditions in ELSA.

Outcomes	Metabolically unhealthy versus healthy status				
	*n*	Model 1		Model 2	
		HR	95% CI	HR	95% CI
Heart disease	4007	1.30	1.10, 1.54**	1.20	0.99, 1.44
Stroke	4774	1.48	1.07, 2.05*	1.45	1.04, 2.03*
Arthritis	3037	1.42	1.20, 1.67***	1.21	1.01, 1.44*
Cancer	4538	0.99	0.81, 1.21	0.93	0.75, 1.15
Memory‐related disease	4895	0.90	0.69, 1.19	0.91	0.68, 1.21

*Note*: Model 1: The association between metabolic status and health outcome was adjusted for age, sex, ethnicity, marital status, paid employment status, completion of degree qualification level, and household wealth. Model 2: Model 1 with an additional adjustment for BMI category.

Abbreviations: n, analytical sample size; HR, hazard ratio; CI, confidence interval.

*p < 0.05; **p < 0.01; ***p < 0.001.

**TABLE 3:** Longitudinal associations between metabolic syndrome status and the development of non‐communicable health conditions in HRS.

Outcomes	Metabolically unhealthy versus healthy status				
	*n*	Model 1		Model 2	
		HR	95% CI	HR	95% CI
Heart disease	6728	1.36	1.19, 1.55***	1.32	1.15, 1.52***
Stroke	8584	1.55	1.28, 1.89***	1.57	1.30, 1.90***
Arthritis	3118	1.04	0.90, 1.21	0.97	0.83, 1.12
Cancer	7831	1.09	0.93, 1.27	1.03	0.88, 1.21
Memory‐related disease	8593	0.97	0.84, 1.11	1.05	0.91, 1.20

*Note*: Model 1: The association between metabolic status and outcome was adjusted for age, sex, ethnicity, marital status, employment status, the number of years in education, and household wealth. Model 2: Model 1 with an additional adjustment for BMI category.

Abbreviations: n, analytical sample size; HR, hazard ratio; CI, confidence interval.

*p < 0.05; **p < 0.01; ***p < 0.001.

**TABLE 4:** Two‐way interaction terms between metabolic syndrome status and psychological measures at baseline in the development of subsequent non‐communicable health conditions in ELSA.

Interaction: Metabolic syndrome (yes vs. no)*psychological measures	*n*	HR	95% CI
Outcome: Heart disease			
Depressive symptoms	4005	0.95	0.79, 1.14
Enjoyment of life	4003	1.13	0.95, 1.33
Eudemonic well‐being	4000	1.21	1.01, 1.44*
Life satisfaction	3973	1.08	0.92, 1.28
Loneliness	4000	1.13	0.96, 1.33
Social support	4005	0.98	0.83, 1.16
Social strain	4003	1.11	0.94, 1.31
Index of psychological distress	4007	0.92	0.78, 1.09
Outcome: Stroke			
Depressive symptoms	4771	0.92	0.66, 1.29
Enjoyment of life	4769	0.99	0.73, 1.35
Eudemonic well‐being	4762	1.02	0.75, 1.40
Life satisfaction	4732	0.96	0.68, 1.36
Loneliness	4761	0.78	0.59, 1.02
Social support	4769	0.99	0.72, 1.36
Social strain	4767	0.88	0.64, 1.21
Index of psychological distress	4774	0.92	0.67, 1.24
Outcome: Arthritis			
Depressive symptoms	3036	1.12	0.94, 1.34
Enjoyment of life	3034	0.82	0.69, 0.97*
Eudemonic well‐being	3030	0.83	0.69, 0.99*
Life satisfaction	3011	0.86	0.72, 1.02
Loneliness	3029	1.23	1.03, 1.48*
Social support	3034	0.93	0.79, 1.09
Social strain	3032	1.01	0.86, 1.19
Index of psychological distress	3037	1.23	1.03, 1.47*
Outcome: Cancer			
Depressive symptoms	4535	0.98	0.77, 1.24
Enjoyment of life	4533	0.90	0.72, 1.13
Eudemonic well‐being	4527	1.03	0.83, 1.28
Life satisfaction	4497	1.09	0.88, 1.36
Loneliness	4526	0.96	0.77, 1.19
Social support	4533	0.98	0.81, 1.19
Social strain	4531	0.88	0.70, 1.11
Index of psychological distress	4538	0.97	0.77, 1.23
Outcome: Memory‐related disease			
Depressive symptoms	4893	0.93	0.73, 1.19
Enjoyment of life	4890	1.10	0.87, 1.39
Eudemonic well‐being	4883	1.38	1.06, 1.81*
Life satisfaction	4850	1.19	0.91, 1.56
Loneliness	4882	1.00	0.79, 1.26
Social support	4890	0.88	0.67, 1.14
Social strain	4888	0.83	0.64, 1.08
Index of psychological distress	4895	0.82	0.65, 1.05

*Note*: The association was adjusted for age, sex, ethnicity, marital status, paid employment status, completion of degree qualification level, household wealth, and BMI category. Index of psychological distress was developed by re‐standardizing the average standardized scores of 5 psychological measures (depressive symptoms, eudemonic wellbeing, enjoyment of life, life satisfaction, loneliness) that were found to load onto a single factor in factor analysis.

Abbreviations: n, analytical sample size; HR, hazard ratio; CI, confidence interval.

*p < 0.05; **p < 0.01; ***p < 0.001.

**TABLE 5:** Two‐way interaction terms between metabolic syndrome status and psychological measures at baseline in the development of subsequent non‐communicable health conditions in HRS.

Interaction: Metabolic syndrome (yes vs. no)*psychological measures	*n*	HR	95% CI
Outcome: Heart disease			
Depressive symptoms	6728	1.05	0.92, 1.20
Life satisfaction	6669	0.88	0.77, 1.01
Loneliness	6652	1.00	0.87, 1.15
Social support	6710	0.92	0.80, 1.06
Social strain	6706	1.01	0.88, 1.16
Positive affect	6658	1.07	0.94, 1.21
Negative affect	6662	0.91	0.80, 1.04
Purpose in life	6621	1.05	0.92, 1.19
Anxiety	6656	0.92	0.80, 1.06
Hopelessness	6688	1.02	0.89, 1.16
Optimism	6648	1.04	0.91, 1.18
Pessimism	6643	1.02	0.89, 1.16
Cynical hostility	6532	1.05	0.91, 1.20
Personal constraint	6685	0.97	0.85, 1.11
Mastery	6688	0.92	0.80, 1.05
Index of psychological distress	6728	0.99	0.86, 1.13
Outcome: Stroke			
Depressive symptoms	8584	1.01	0.85, 1.20
Life satisfaction	8512	0.89	0.74, 1.07
Loneliness	8495	0.93	0.76, 1.14
Social support	8563	1.11	0.93, 1.31
Social strain	8558	1.06	0.89, 1.27
Positive affect	8504	1.09	0.90, 1.31
Negative affect	8508	0.97	0.82, 1.14
Purpose in life	8448	1.10	0.92, 1.31
Anxiety	8492	1.16	0.96, 1.40
Hopelessness	8539	0.83	0.69, 1.01
Optimism	8495	0.96	0.80, 1.15
Pessimism	8494	0.95	0.79, 1.13
Cynical hostility	8343	0.93	0.76, 1.14
Personal constraint	8524	0.91	0.76, 1.10
Mastery	8530	0.97	0.81, 1.16
Index of psychological distress	8584	0.95	0.79, 1.15
Outcome: Arthritis			
Depressive symptoms	3118	1.05	0.91, 1.22
Life satisfaction	3093	1.03	0.89, 1.18
Loneliness	3088	1.00	0.86, 1.16
Social support	3109	0.98	0.85, 1.13
Social strain	3108	0.87	0.75, 1.00
Positive affect	3096	0.88	0.76, 1.01
Negative affect	3099	0.99	0.85, 1.14
Purpose in life	3075	1.00	0.86, 1.16
Anxiety	3085	0.94	0.80, 1.10
Hopelessness	3101	1.05	0.91, 1.22
Optimism	3089	0.99	0.85, 1.15
Pessimism	3087	1.06	0.92, 1.23
Cynical hostility	3030	0.97	0.83, 1.12
Personal constraint	3100	1.01	0.87, 1.17
Mastery	3106	1.00	0.87, 1.14
Index of psychological distress	3118	1.04	0.90, 1.21
Outcome: Cancer			
Depressive symptoms	7831	1.12	0.95, 1.31
Life satisfaction	7761	0.98	0.84, 1.13
Loneliness	7741	0.83	0.71, 0.98*
Social support	7810	1.02	0.87, 1.19
Social strain	7806	0.97	0.83, 1.13
Positive affect	7746	0.93	0.81, 1.08
Negative affect	7754	1.01	0.86, 1.17
Purpose in life	7709	1.01	0.87, 1.17
Anxiety	7738	1.04	0.88, 1.22
Hopelessness	7788	1.00	0.86, 1.17
Optimism	7744	1.01	0.86, 1.18
Pessimism	7740	0.98	0.84, 1.14
Cynical hostility	7601	0.97	0.83, 1.13
Personal constraint	7777	1.05	0.90, 1.22
Mastery	7779	0.87	0.74, 1.01
Index of psychological distress	7831	1.01	0.87, 1.17
Outcome: Memory‐related disease			
Depressive symptoms	8593	0.95	0.84, 1.07
Life satisfaction	8524	0.91	0.80, 1.05
Loneliness	8510	0.99	0.87, 1.13
Social support	8576	0.84	0.73, 0.96*
Social strain	8572	1.03	0.90, 1.18
Positive affect	8514	1.06	0.93, 1.22
Negative affect	8516	0.86	0.75, 0.98*
Purpose in life	8466	1.09	0.95, 1.24
Anxiety	8506	0.91	0.78, 1.04
Hopelessness	8548	1.04	0.91, 1.19
Optimism	8508	0.99	0.87, 1.13
Pessimism	8503	1.07	0.93, 1.22
Cynical hostility	8358	0.98	0.84, 1.13
Personal constraint	8534	0.97	0.86, 1.10
Mastery	8539	1.07	0.94, 1.21
Index of psychological distress	8593	0.95	0.84, 1.09

*Note*: The association was adjusted for age, sex, ethnicity, marital status, employment status, the number of years in education, household wealth, and BMI category. Index of psychological distress was developed by re‐standardizing the average standardized scores of 10 psychological measures (depressive symptoms, life satisfaction, loneliness, positive affect, negative affect, purpose in life, anxiety, hopelessness, pessimism, and personal constraint) that were found to load onto a single factor in factor analysis.

Abbreviations: n, analytical sample size; HR, hazard ratio; CI, confidence interval.

*p < 0.05; **p < 0.01; ***p < 0.001.

## 4 DISCUSSION

Across two large nationally representative studies of UK and US older adults, there was convincing evidence that MetSyn was associated with a considerable psychological burden. These findings replicate previous research identifying cross‐sectional relationships between MetSyn and both depression and anxiety.^5^, ^17^ In addition, MetSyn was associated with a range of other negative psychosocial factors, including hopelessness, cynical hostility, increased negative affect, decreased positive affect, social support and purpose in life.^19^, ^20^ Unlike in previous research, a global approach to considering the psychological burden associated with MetSyn was adopted by examining how MetSyn is related to a wide range of psychological factors. The present findings suggest that MetSyn is characterized by a clustering of numerous negative psychological variables including mental health (e.g., anxiety, depression), life outlook (e.g., purpose in life, pessimism) and social (e.g., perceived social support and social strain) factors. An implication of these findings is that the considerable psychological burden associated with MetSyn would benefit from being addressed (e.g., through access to psychological therapies) and/or considered in the clinical management and treatment of patients with MetSyn.^14^

As expected, MetSyn was predictive of an increased risk of developing heart disease and stroke.^1^ However, there was no convincing evidence that the increased risk of developing NCDs associated with MetSyn was reliably moderated or mediated by psychological factors. Based on these findings, MetSyn and its associated psychological burden are therefore likely to have independent and additive effects on NCD risk, as opposed to psychological factors explaining why MetSyn increases NCD development risk. These findings are consistent with other research using HRS and ELSA, which suggests that the direct relationship between obesity and NCD risk is also not explained by people with obesity having lower psychological well‐being than people without obesity.^18^

Limitations of the present work are that findings may not generalize to younger adults and due to available data, definitions of MetSyn differed between UK and US samples. However, the results were comparable between the two. Future research will benefit from better understanding why psychosocial measures are associated with MetSyn, as it is feasible that they may predispose individuals to the development of MetSyn and/or be a direct consequence of MetSyn. These limitations notwithstanding, the present study findings highlight the need to monitor and intervene to improve psychosocial well‐being among individuals living with MetSyn. Related to this, future research may benefit from examining how the psychological burden associated with MetSyn impacts management and treatment outcomes associated with MetSyn.^21^

## AUTHOR CONTRIBUTIONS

**Eric Robinson**: conceptualization; methodology; project administration; supervision; funding acquisition; writing – original draft; review and editing. **Michael Daly**: conceptualization; methodology; funding acquisition; writing – review and editing. **I Gusti Ngurah Edi Putra**: conceptualization; methodology; project administration; data curation; formal analysis; visualization; writing – original draft; review and editing.

## CONFLICT OF INTEREST STATEMENT

The authors declare that there are no competing interests.

## Supporting information

Supporting Information S1

## Data Availability

Data are available online (ELSA: https://doi.org/10.5255/UKDA‐SN‐5050‐25; HRS: https://hrsdata.isr.umich.edu/data‐products/rand) with the permission of the data custodians.
