# Supplementary material for: The psychological burden associated with metabolic syndrome: Evidence from UK and US older adults
Source: Obes Sci Pract. 2024 Jul 6;10(4):e780. doi: 10.1002/osp4.780 (PMC11227276; doi:10.1002/osp4.780)
Supplement: Supplementary file 1 — Supporting Information S1 [file OSP4-10-e780-s001.pdf]

Online Supplementary Materials

Page 2-4: Psychological measures examined, including scale information, example items and scoring in ELSA and HRS samples

Page 5: Non-communicable disease measurement

Page 6: Additional analysis information for handling of missing data and mediation analyses

Page 7: Participant characteristics in ELSA and HRS samples

Page 8-11: Psychological measures and risk of NCD development analyses

Page 12-13: Analyses examining if psychological measures mediate associations between metabolic syndrome and risk of NCD development

Page 14-15: References

Psychological measures in ELSA and HRS

1. Depressive symptoms

Depressive symptoms were assessed using an 8-item version of the Center for Epidemiological Studies-Depression (CES-D)[1] in which participants reported the extent to which they experienced negative feelings in the last seven days (e.g., "*Did you feel depressed?*"). Participants' responses ("*yes*" = 1 and "*no*" = 0) were summed to generate a summary score on a scale of 0 to 8. A higher score was indicative of higher level of depressive symptoms. This scale had good internal consistency in both studies (Cronbach's alpha = 0.81 in ELSA and 0.82 in HRS).

2. Enjoyment of life

Following previous studies,[2-4] enjoyment of life was evaluated based on four items (e.g., *"I enjoy being in the company of others"*) from the quality of life questionnaire of Control, Autonomy, Self-Realization and Pleasure (CASP-19).[5] We reversed coded participants' responses on a 4-point Likert scale (from 0= "*often*" to 3= "*never*") and then added them together. Greater enjoyment of life was shown by a higher total score on a scale of 0 to 12. This measure was found to have acceptable internal consistency in ELSA (Cronbach's alpha = 0.70).

3. Eudemonic well-being

As was informed by a previous approach,[4,6] the remaining 15 items from CASP-19[5] were used to construct eudemonic well-being (e.g., *"How often feels what happens to them is out of their control"*). With a similar scoring system as enjoyment of life, a higher total score ranging from 0 to 45 indicated better eudemonic well-being. In ELSA, this scale had good internal consistency (Cronbach's alpha = 0.86).

4. Life satisfaction

The Satisfaction with Life Scale (SWLS)[7] consisting of five items (e.g., "*In most ways my life is close to my ideal"*) with a 7-point Likert scale (1= "*strongly disagree"* to 7= "*strongly agree")* was used to assess the level of life satisfaction. Responses to all the items were added together, resulting in a summary score on a scale of 1 to 35 with a higher score indicating a higher level of life satisfaction. This measure had good internal consistency (Cronbach's alpha = 0.90 in ELSA and 0.89 in HRS).

5. Loneliness

As was informed by a past study,[4,8] a three-item loneliness scale [9] was used to determine loneliness. Participants rated on a 3-point Likert scale (1= "*hardly ever or never*," 2= "*some of the time*," and 3= "*often*") to the following questions: *"How often do you feel: you lack companionship?", "isolated from others?", "left out?").* A higher score on a total score ranging from 1 to 9 indicated greater loneliness. In both studies, a three-item loneliness scale was observed to have good internal consistency (Cronbach's alpha = 0.82 in ELSA and 0.81 in HRS).

6. Social support

Participants were asked three questions (e.g., "*How much do they really understand the way you feel about things?"*) regarding relationships they had with different people, such as

partner, children, other immediate family members, and friends. Their responses on a 4-point Likert scale (1= "*a lot*" to 4= "*not at all*") were reversed-coded and averaged (e.g., as in [4,10]). To assess overall social support, we re-averaged the social support scores received from different reported relationships. A higher summary score on a scale of 1 to 4 indicated better social support. Cronbach's alpha for this measure was 0.53 in ELSA and 0.56 in HRS.

7. Social strain

Similar to the scoring system of social support, participants' responses to three items (e.g., "*How much do they criticise you?*") on a 4-point-Likert scale (1= "*a lot*" to 4= "*not at all*") (e.g., as in [4,10]) for each different relationship were reverse coded and averaged. An overall social strain summary score was determined by re-averaging the scores reported from different relationships. A higher summary score on a scale of 1 to 4 indicated higher social strain. This scale has acceptable internal consistency (Cronbach's alpha = 0.69 in ELSA and 0.71 in HRS).

8. Positive affect

We used 13 5-point Likert-scale (1= "*not at all*" to 5= "*very much*") items from the Positive and Negative Affect scale (PANAS-X)[11] to quantify positive affect. Participants' responses on how they felt in the last month (e.g., "*During the last 30 days, to what degree did you feel enthusiastic?*") were totalled to create a summary score ranging from 1 to 65. A higher summary score was indicative of a greater level of positive affect. This scale to measure positive affect in HRS was observed to have very good internal consistency (Cronbach's alpha = 0.92)

9. Negative affect

Similar to the scoring system of positive affect, the remaining 12 5-point Likert-scale items from PANAS-X [11] determined negative affect (e.g., "*During the last 30 days, to what degree did you feel upset?*"). A higher score on a possible range of 1-to-60 for a total score indicated a greater level of negative affect. Cronbach's alpha of 0.90 was observed for the scale measuring negative affect in HRS, indicating very good internal consistency.

10. Purpose in life

We evaluated purpose in life using the purpose in life domain from the Ryff Measures of Psychological Wellbeing.[12,13] Participants responded to seven 6-point-Likert-scale items (e.g., "*I enjoy making plans for the future and working to make them a reality.*") (1= "*strongly disagree*" to 6= "*strongly agree*"). To create a summary score, responses to all the items were totalled. A higher level of purpose in life was shown by a higher score on a scale of 1 to 42. The purpose in life scale was found to have acceptable internal consistency in HRS (Cronbach's alpha = 0.77).

11. Anxiety

Anxiety was evaluated using the Beck Anxiety Inventory (BAI).[14] Participants rated the extent to which they felt negative emotions in the last seven days (e.g., "*I had fear of the worst happening*"). Responses to all the items (n = 5) on a 4-point Likert scale (1= *"never"* to 4= *"most of the time"*) were totalled to create a summary score on a scale of 1 to 20 with a higher score indicating greater anxiety. The five-item scale to measure anxiety in HRS had good internal consistency (Cronbach's alpha = 0.82).

12. Hopelessness

We used four items from two different scales to assess hopelessness: two items from Everson, Kaplan [15] and two other items from Beck, Weissman [16] (e.g., "*I feel it is impossible for me to reach the goals that I would like to strive for.*", "*I don't expect to get what I really want.*"). Responses on a 6-point Likert scale (1= "*strongly disagree*" to 6= "*strongly agree*") were added together to develop a total score on a scale of 1 to 24. Greater hopelessness was shown by a higher score. Cronbach's alpha for this scale was 0.85 in HRS, indicative of good internal consistency.

13. Optimism

Three 6-point Likert-scale items from the Life Orientation Test-Revised (LOT-R) [17] were used to quantify optimism (e.g., "*I'm always optimistic about my future.*") (1= "*strongly disagree*" to 6= "*strongly agree*"). A total score on a scale of 1 to 18 was calculated by summing responses to all the items. A higher score was indicative of better optimism. Good internal consistency was observed for this scale in HRS (Cronbach's alpha = 0.80).

14. Pessimism

We assessed pessimism using the other three 6-point Likert-scale items from the LOT-R (e.g., "*If something can go wrong for me it will.*").[17] With a similar scoring system to optimism, a summary score on a scale of 1 to 18 was calculated with a higher score indicating a higher level of pessimism. This scale also had acceptable internal consistency in HRS (Cronbach's alpha = 0.77).

15. Cynical hostility

Participants were asked to complete the Cook-Medley Hostility Inventory to assess their level of cynical hostility. This scale consists of five 6-point Likert-scale items (e.g., "*Most people dislike putting themselves out to help other people.*") (1= "*strongly disagree*" to 6= "*strongly agree*").[4,18,19] To construct a summary score, all the responses were summed, resulting in a total score on a scale of 1 to 30 with a higher score indicating a higher level of cynical hostility. This measure had acceptable internal consistency in HRS (Cronbach's alpha = 0.79).

16. Personal constraints

Personal constraints were evaluated by asking participants to rate the extent to which they perceived constraints over their life conditions. They responded to five 6-point Likert-scale items (e.g., "*I often feel helpless in dealing with the problems of life.*") (1= "*strongly disagree*" to 6= "*strongly agree*").[4,20] A summary score was generated by adding together responses to all the items. A higher summary score on a scale from 1 to 30 indicated more perceived personal constraints. This scale was observed to have good internal consistency in HRS (Cronbach's alpha = 0.86).

17. Mastery

Perceived mastery was evaluated using five 6-point Likert scale (1= "*strongly disagree*" to 6= "*strongly agree*") items (e.g., "*I can do just about anything I really set my mind to.*").[4,20] A total score ranging from 1 to 30 was computed by adding together responses to all the items. A higher score was indicative of better perceived mastery. In HRS, this mastery scale was found to have Cronbach's alpha of 0.90, indicating very good internal consistency.

NCD measurement in ELSA and HRS

NCDs	ELSA	HRS
Heart diseases	Self-reported diagnosis<br>"Has a doctor ever told that (or had) any of the conditions on this card?" "Angina", "A heart attack (including myocardial infarction or coronary, thrombosis)", "Congestive heart failure", "A heart murmur", "An abnormal heart rhythm", "Any other heart trouble"	Self-reported diagnosis<br>"Has a doctor ever told you that you had a heart attack, coronary heart disease, angina, congestive heart failure, or other heart problems?"
Stroke	Self-reported diagnosis<br>"Has a doctor ever told that (or had) any of the conditions on this card?" "A stroke (cerebral vascular disease)"	Self-reported diagnosis<br>"Has a doctor ever told you that you had a stroke?"
Arthritis	Self-reported diagnosis<br>"Has a doctor ever told that (or had) any of the conditions on this card?" "Arthritis"	Self-reported diagnosis<br>"Have you ever had, or has a doctor ever told you that you have arthritis or rheumatism?"
Cancer	Self-reported diagnosis<br>"Has a doctor ever told that (or had) any of the conditions on this card?" "Cancer or a malignant tumour"	Self-reported diagnosis<br>"Has a doctor ever told you that you have cancer or a malignant tumor, excluding minor skin cancer?"
Memory-related diseases	Self-reported diagnosis<br>"Has a doctor ever told that (or had) any of the conditions on this card?" "Dementia, senility or any other serious memory impairment"<br>– OR –<br>Interview<br>A total score of ≤ 6 (out of 27) was used to define probable dementia using the 27-point modified Telephone Interview for Cognitive Status.[21-23]<br>– OR –<br>An average score of ≥ 3.5 (out of 5) defined probable dementia using a short-form 16-item Informant Questionnaire on Cognitive Decline in the Elderly (IQCODE).[10,24]	Self-reported diagnosis<br>"Has a doctor ever told you that you have a memory-related disease?"<br>– OR –<br>Interview<br>A total score of ≤ 6 (out of 27) was used to define probable dementia using the 27-point modified Telephone Interview for Cognitive Status.[21-23]<br>– OR –<br>An average score of ≥ 3.5 (out of 5) defined probable dementia using a short-form 16-item Informant Questionnaire on Cognitive Decline in the Elderly (IQCODE).[10,24]

We updated our pre-registered study protocol (https://doi.org/10.17605/OSF.IO/JRQAP) by including additional assessment for dementia using the short-form IQCODE questionnaire.

This data is drawn from the same sample of participants as in https://doi.org/10.1038/s41366-024-01551-1. Table data and/or information is therefore the same.

Plan for mediation analyses and missing data

We used the 'med4way' in STATA [25] to perform causal mediation analysis to examine the role of psychological measures in explaining (mediating) the longitudinal association between baseline metabolic syndrome (MetSyn) and risk of developing non-communicable diseases (NCDs). We fitted separate single mediation models for mediation by each psychological measure, controlling for age, sex, ethnicity, marital status, employment status, education, household wealth, and BMI category. Similar to regression models, our mediation models for each NCD also excluded participants with that NCD of interest reported at baseline or before. Using med4way, we calculated the proportion mediated (i.e., "overall proportion due to mediation"), defined by summing proportions of pure indirect effect and mediated interaction.[4,26]

In med4way, mediation was determined by fitting two regression models: 1) the exposure (independent variable, MetSyn) on the mediator (psychological measures), and 2) the exposure on the outcome (dependent variable, NCDs), accounting for and in interaction with the mediator. Linear regression was selected for a model of MetSyn on psychological measures. Meanwhile, we used an accelerated failure time model for regression models of MetSyn on NCDs (e.g., as in [4,27]) as bias estimates are more likely to be produced when Cox regression is used in mediation analyses with common or non-rare outcomes.[28,29]

As we found missing observations across studies and sociodemographic characteristics (e.g., age, sex, ethnicity) were predictive of missingness, an inverse probability weighting approach was used to minimise potential selection bias.[30,31] We fitted a logistic regression model to examine sociodemographic characteristics associated with having complete information or being included in the analysis. The probability of being retained from the logistic regression model was then computed and inversed to create new sample weights that compensate for differences in characteristics of being included in the analysis. Next, these sample weights were multiplied with baseline non-response weights to account for differences in characteristics associated with non-response at baseline.

Characteristics of the participants

Characteristics of the participants in ELSA and HRS

	ELSA			HRS		
	n[a]	Mean (SD)[b]	%[b]	n[a]	Mean (SD)[b]	%[b]
**Sociodemographic characteristics at baseline**						
Age (years)	8,127	65.92 (10.67)		12,477	65.99 (10.15)	
Sex	8,127			12,477		
Female			53.45			54.85
Male			46.55			45.15
Ethnicity	8,124			12,472		
Non-White			3.76			13.09
White			96.24			86.91
BMI baseline (in kg/m^2)	7,768	28.44 (5.30)		10,906	29.65 (5.98)	
Normal weight			25.97			21.67
Overweight			41.79			36.37
Class I obesity			21.54			25.05
Class II & III obesity			10.70			16.91
Metabolic syndrome	6,067			11,211		
No		40.03				47.56
Yes		59.97				52.44
**Eligible participants and the incidence of follow-up NCDs[c]**						
Heart disease	5,891			8,404		
Yes			17.42			18.20
Stroke	7,168			10,719		
Yes			4.61			6.91
Arthritis	4,530			3,889		
Yes			21.28			33.16
Cancer	6,848			9,820		
Yes			9.08			11.99
Memory disease	7,422			10,773		
Yes			7.69			11.12

[a] Participants without missing observations were included in the calculations.

[b] Weighted values were calculated using sample weights from physical assessments.

[c] Participants with a corresponding NCD reported before and at the baseline were excluded.

This data is drawn from the same sample of participants as in https://doi.org/10.1038/s41366-024-01551-1. Table data and/or information is therefore the same.

Psychological measures and risk of NCD development results

Longitudinal associations between psychological measures and the development of non-communicable health conditions in ELSA

	n	HR	95% CI
**Outcome: Heart disease**			
Depressive symptoms	5,080	1.11	1.03, 1.20**
Enjoyment of life	5,076	0.90	0.84, 0.96**
Eudemonic well-being	5,073	0.88	0.82, 0.95**
Life satisfaction	5,035	0.89	0.83, 0.95**
Loneliness	5,071	1.11	1.04, 1.19**
Social support	5,077	0.94	0.88, 1.01
Social strain	5,075	1.05	0.97, 1.12
Index of psychological distress	5,082	1.16	1.08, 1.25***
**Outcome: Stroke**			
Depressive symptoms	6,178	1.10	0.97, 1.24
Enjoyment of life	6,173	0.88	0.79, 0.98*
Eudemonic well-being	6,166	0.83	0.74, 0.95**
Life satisfaction	6,125	0.93	0.81, 1.06
Loneliness	6,160	1.04	0.92, 1.18
Social support	6,175	0.94	0.83, 1.07
Social strain	6,173	0.97	0.85, 1.11
Index of psychological distress	6,181	1.15	1.02, 1.29*
**Outcome: Arthritis**			
Depressive symptoms	3,928	1.17	1.08, 1.26***
Enjoyment of life	3,925	0.85	0.79, 0.91***
Eudemonic well-being	3,921	0.80	0.74, 0.86***
Life satisfaction	3,897	0.82	0.76, 0.88***
Loneliness	3,917	1.13	1.05, 1.22**
Social support	3,924	0.88	0.82, 0.94***
Social strain	3,922	1.09	1.01, 1.17*
Index of psychological distress	3,929	1.26	1.17, 1.36***
**Outcome: Cancer**			
Depressive symptoms	5,867	1.06	0.96, 1.18
Enjoyment of life	5,862	0.97	0.88, 1.07
Eudemonic well-being	5,856	1.01	0.91, 1.11
Life satisfaction	5,815	0.94	0.85, 1.04
Loneliness	5,850	1.02	0.92, 1.13
Social support	5,863	0.99	0.90, 1.07
Social strain	5,861	1.05	0.96, 1.16

Index of psychological distress	5,870	1.05	0.94, 1.16
**Outcome: Memory-related disease**			
Depressive symptoms	6,374	1.27	1.15, 1.41***
Enjoyment of life	6,368	0.75	0.68, 0.82***
Eudemonic well-being	6,361	0.68	0.62, 0.76***
Life satisfaction	6,315	0.82	0.74, 0.92**
Loneliness	6,353	1.23	1.11, 1.36**
Social support	6,368	0.84	0.76, 0.94**
Social strain	6,366	1.18	1.06, 1.32**
Index of psychological distress	6,376	1.43	1.29, 1.58***

**p<0.05; **p<0.01; ***p<0.001*

n=analytical sample size; HR=hazard ratio; CI=confidence interval

The association was controlled for age, sex, ethnicity, marital status, employment status, education, household wealth, and BMI category.

Index of psychological distress was developed by re-standardizing the average standardized scores of 5 psychological measures (depressive symptoms, eudemonic wellbeing, enjoyment of life, life satisfaction, loneliness) that were found to load onto a single factor in factor analysis.

This data is drawn from the same sample of participants as in https://doi.org/10.1038/s41366-024-01551-1. Table data and/or information is therefore the same.

Longitudinal associations between psychological measures and the development of non-communicable health conditions in HRS

	**n**	**HR**	**95% CI**
**Outcome: Heart disease**			
Depressive symptoms	7,443	1.13	1.06, 1.21***
Life satisfaction	7,380	0.86	0.80, 0.92***
Loneliness	7,359	1.08	1.01, 1.16*
Social support	7,423	0.92	0.86, 0.98*
Social strain	7,419	1.03	0.97, 1.11
Positive affect	7,370	0.95	0.89, 1.01
Negative affect	7,373	1.10	1.03, 1.17**
Purpose in life	7,325	0.96	0.90, 1.02
Anxiety	7,354	1.14	1.07, 1.23***
Hopelessness	7,398	1.08	1.01, 1.15*
Optimism	7,353	0.95	0.89, 1.01
Pessimism	7,350	1.09	1.02, 1.16**
Cynical hostility	7,229	1.01	0.95, 1.09

Personal constraint	7,391	1.09	1.03, 1.16**
Mastery	7,394	0.95	0.89, 1.01
Index of psychological distress	7,443	1.15	1.07, 1.23***
**Outcome: Stroke**			
Depressive symptoms	9,440	1.16	1.07, 1.26***
Life satisfaction	9,361	0.84	0.77, 0.92***
Loneliness	9,339	1.15	1.05, 1.27**
Social support	9,416	0.92	0.84, 0.99*
Social strain	9,411	1.16	1.06, 1.26**
Positive affect	9,356	0.92	0.84, 0.99*
Negative affect	9,359	1.20	1.11, 1.30***
Purpose in life	9,292	0.85	0.78, 0.92***
Anxiety	9,331	1.25	1.15, 1.37***
Hopelessness	9,389	1.19	1.09, 1.31***
Optimism	9,339	0.93	0.85, 1.01
Pessimism	9,339	1.17	1.07, 1.28***
Cynical hostility	9,175	1.13	1.03, 1.25*
Personal constraint	9,371	1.15	1.05, 1.25**
Mastery	9,377	0.95	0.87, 1.03
Index of psychological distress	9,440	1.29	1.18, 1.41***
**Outcome: Arthritis**			
Depressive symptoms	3,486	1.23	1.14, 1.32***
Life satisfaction	3,461	0.85	0.79, 0.91***
Loneliness	3,456	1.12	1.04, 1.21**
Social support	3,477	0.95	0.88, 1.02
Social strain	3,476	1.15	1.07, 1.24***
Positive affect	3,463	0.87	0.81, 0.93***
Negative affect	3,465	1.18	1.09, 1.26***
Purpose in life	3,439	0.88	0.82, 0.95**
Anxiety	3,448	1.18	1.09, 1.27***
Hopelessness	3,468	1.17	1.09, 1.26***
Optimism	3,452	0.94	0.87, 1.01
Pessimism	3,452	1.14	1.06, 1.23***
Cynical hostility	3,388	1.08	0.99, 1.16
Personal constraint	3,465	1.06	0.98, 1.14
Mastery	3,473	0.95	0.89, 1.02
Index of psychological distress	3,486	1.24	1.15, 1.34***
**Outcome: Cancer**			
Depressive symptoms	8,609	1.05	0.97, 1.13
Life satisfaction	8,531	0.94	0.87, 1.01
Loneliness	8,509	1.02	0.94, 1.10

Social support	8,585	0.99	0.92, 1.07
Social strain	8,581	0.98	0.91, 1.06
Positive affect	8,519	0.96	0.89, 1.03
Negative affect	8,526	0.99	0.91, 1.06
Purpose in life	8,473	1.01	0.93, 1.08
Anxiety	8,497	1.00	0.92, 1.08
Hopelessness	8,562	1.08	0.99, 1.17
Optimism	8,513	0.98	0.92, 1.06
Pessimism	8,509	1.07	0.99, 1.16
Cynical hostility	8,359	1.01	0.93, 1.09
Personal constraint	8,546	0.99	0.92, 1.07
Mastery	8,550	0.98	0.91, 1.05
Index of psychological distress	8,609	1.04	0.97, 1.13
**Outcome: Memory-related disease**			
Depressive symptoms	9,462	1.28	1.21, 1.36***
Life satisfaction	9,386	0.89	0.83, 0.95**
Loneliness	9,368	1.28	1.20, 1.37***
Social support	9,442	0.92	0.86, 0.99*
Social strain	9,438	1.19	1.11, 1.28***
Positive affect	9,378	0.83	0.77, 0.89***
Negative affect	9,379	1.21	1.13, 1.30***
Purpose in life	9,322	0.82	0.76, 0.87***
Anxiety	9,359	1.31	1.22, 1.41***
Hopelessness	9,412	1.28	1.20, 1.37***
Optimism	9,367	0.88	0.83, 0.94***
Pessimism	9,363	1.26	1.18, 1.35***
Cynical hostility	9,205	1.19	1.11, 1.28***
Personal constraint	9,393	1.27	1.20, 1.35***
Mastery	9,398	0.90	0.85, 0.96**
Index of psychological distress	9,462	1.41	1.32, 1.51***

**p<0.05; **p<0.01; ***p<0.001*

n=analytical sample size; HR=hazard ratio; CI=confidence interval

The association was controlled for age, sex, ethnicity, marital status, employment status, education, household wealth, and BMI category.

Index of psychological distress was developed by re-standardizing the average standardized scores of 10 psychological measures (depressive symptoms, life satisfaction, loneliness, positive affect, negative affect, purpose in life, anxiety, hopelessness, pessimism, and personal constraint) that were found to load onto a single factor in factor analysis.

This data is drawn from the same sample of participants as in https://doi.org/10.1038/s41366-024-01551-1. Table data and/or information is therefore the same.

Mediation of metabolic syndrome-NCD risk by psychological measures results

Formal testing of mediation of metabolic syndrome (MetSyn)-NCD risk by psychological variables was conducted if pre-conditions of mediation were met,[32] which were that: MetSyn status was associated with i) psychological measure and ii) NCD, and iii) psychological measure was associated with NCD.

Mediation by psychological measures in the longitudinal association between metabolic syndrome status and development of non-communicable health conditions in ELSA

	**Mediation by psychological measures**		
	**Metabolic syndrome (yes vs. no)**		
	**n**	**Estimate**	**95% CI**
**Outcome: Stroke**			
Enjoyment of life	4,769	0.043	-0.029, 0.114
Eudemonic well-being	4,762	0.042	-0.020, 0.103
Index of psychological distress	4,774	0.030	-0.020, 0.081
**Outcome: Arthritis**			
Depressive symptoms	3,036	0.067	-0.041, 0.175
Enjoyment of life	3,034	0.182	-0.068, 0.431
Eudemonic well-being	3,030	0.175	-0.096, 0.447
Loneliness	3,029	0.042	-0.068, 0.152
Social support	3,034	0.060	-0.035, 0.155
Social strain	3,032	0.031	-0.027, 0.090
Index of psychological distress	3,037	0.194	-0.115, 0.503

**p<0.05; **p<0.01; ***p<0.001*

n=analytical sample size; Estimate=the overall proportion due to mediation or the proportion mediated; CI=confidence interval

The mediation model was controlled for age, sex, ethnicity, marital status, employment status, education, household wealth, and BMI category.

Index of psychological distress was developed by re-standardizing the average standardized scores of 5 psychological measures (depressive symptoms, eudemonic wellbeing, enjoyment of life, life satisfaction, loneliness) that were found to load onto a single factor in factor analysis.

Mediation by psychological measures in the longitudinal association between metabolic syndrome status and the development of non-communicable health conditions in HRS

	**Mediation by psychological measures**		
	**Metabolic syndrome (yes vs. no)**		
	**n**	**Estimate**	**95% CI**
**Outcome: Heart disease**			
Depressive symptoms	6,728	0.017	-0.007, 0.041
Life satisfaction	6,669	0.018	-0.017, 0.052
Loneliness	6,652	0.012	-0.008, 0.032
Social support	6,710	0.031	-0.004, 0.066
Negative affect	6,662	0.005	-0.007, 0.016
Anxiety	6,656	0.015	-0.007, 0.038
Hopelessness	6,688	0.014	-0.007, 0.035
Pessimism	6,643	0.019	-0.008, 0.046
Personal constraint	6,685	0.011	-0.007, 0.029
Index of psychological distress	6,728	0.026	-0.004, 0.056
**Outcome: Stroke**			
Depressive symptoms	8,584	0.019	-0.001, 0.038
Life satisfaction	8,512	0.029	-0.001, 0.059
Loneliness	8,495	0.011	-0.005, 0.027
Social support	8,563	0.009	-0.006, 0.024
Social strain	8,558	0.022	-0.001, 0.045
Positive affect	8,504	0.008	-0.008, 0.023
Negative affect	8,508	0.014	-0.004, 0.032
Purpose in life	8,448	0.018	-0.003, 0.038
Anxiety	8,492	0.042	0.003, 0.082*
Hopelessness	8,539	0.014	-0.003, 0.030
Pessimism	8,494	0.018	-0.002, 0.039
Cynical hostility	8,343	0.015	-0.006, 0.037
Personal constraint	8,524	0.012	-0.004, 0.028
Index of psychological distress	8,584	0.040	0.007, 0.073*

**p<0.05; **p<0.01; ***p<0.001*

n=analytical sample size; Estimate=the overall proportion due to mediation or the proportion mediated; CI=confidence interval

The mediation model was controlled for age, sex, ethnicity, marital status, employment status, education, household wealth, and BMI category.

Index of psychological distress was developed by re-standardizing the average standardized scores of 10 psychological measures (depressive symptoms, life satisfaction, loneliness, positive affect, negative affect, purpose in life, anxiety, hopelessness, pessimism, and personal constraint) that were found to load onto a single factor in factor analysis.

References

1. Turvey CL, Wallace RB, Herzog R. A revised CES-D measure of depressive symptoms and a DSM-based measure of major depressive episodes in the elderly. *International psychogeriatrics* 1999; **11**(2): 139-48.
2. Steptoe A, Wardle J. Enjoying life and living longer. *Arch Intern Med* 2012; **172**(3): 273-5.
3. Zaninotto P, Wardle J, Steptoe A. Sustained enjoyment of life and mortality at older ages: analysis of the English Longitudinal Study of Ageing. *BMJ* 2016; **355**: i6267.
4. Putra IGNE, Daly M, Sutin A, Steptoe A, Scholes S, Robinson E. Obesity, psychological well-being related measures, and risk of seven non-communicable diseases: evidence from longitudinal studies of UK and US older adults. *International Journal of Obesity* 2024.
5. Hyde M, Wiggins RD, Higgs P, Blane DB. A measure of quality of life in early old age: The theory, development and properties of a needs satisfaction model (CASP-19). *Aging & Mental Health* 2003; **7**(3): 186-94.
6. Steptoe A, Demakakos P, de Oliveira C. The psychological well-being, health and functioning of older people in England. *The dynamics of ageing* 2012: 98.
7. Diener E, Emmons RA, Larsen RJ, Griffin S. The Satisfaction With Life Scale. *Journal of Personality Assessment* 1985; **49**(1): 71-5.
8. Davies K, Maharani A, Chandola T, Todd C, Pendleton N. The longitudinal relationship between loneliness, social isolation, and frailty in older adults in England: a prospective analysis. *The Lancet Healthy Longevity* 2021; **2**(2): e70-e7.
9. Hughes ME, Waite LJ, Hawkley LC, Cacioppo JT. A Short Scale for Measuring Loneliness in Large Surveys: Results From Two Population-Based Studies. *Research on aging* 2004; **26**(6): 655-72.
10. Khondoker M, Rafnsson SB, Morris S, Orrell M, Steptoe A. Positive and Negative Experiences of Social Support and Risk of Dementia in Later Life: An Investigation Using the English Longitudinal Study of Ageing. *Journal of Alzheimer's Disease* 2017; **58**: 99-108.
11. Watson D, Clark LA. The PANAS-X: Manual for the Positive and Negative Affect Schedule - Expanded Form: University of Iowa, 1994.
12. Ryff CD. Happiness is everything, or is it? Explorations on the meaning of psychological well-being. *Journal of personality and social psychology* 1989; **57**(6): 1069-81.
13. Ryff CD, Keyes CL. The structure of psychological well-being revisited. *J Pers Soc Psychol* 1995; **69**(4): 719-27.
14. Beck AT, Epstein N, Brown G, Steer RA. An inventory for measuring clinical anxiety: psychometric properties. *J Consult Clin Psychol* 1988; **56**(6): 893-7.
15. Everson SA, Kaplan GA, Goldberg DE, Salonen R, Salonen JT. Hopelessness and 4-year progression of carotid atherosclerosis. The Kuopio Ischemic Heart Disease Risk Factor Study. *Arterioscler Thromb Vasc Biol* 1997; **17**(8): 1490-5.
16. Beck AT, Weissman A, Lester D, Trexler L. The measurement of pessimism: The Hopelessness Scale. *Journal of Consulting and Clinical Psychology* 1974; **42**(6): 861-5.
17. Scheier MF, Carver CS, Bridges MW. Distinguishing optimism from neuroticism (and trait anxiety, self-mastery, and self-esteem): a reevaluation of the Life Orientation Test. *J Pers Soc Psychol* 1994; **67**(6): 1063-78.

18. Cook WW, Medley DM. Proposed hostility and Pharisaic-virtue scales for the MMPI. *Journal of Applied Psychology* 1954; **38**(6): 414-8.
19. Costa PT, Zonderman AB, McCrae RR, Williams RB. Cynicism and paranoid alienation in the Cook and Medley HO Scale. *Psychosomatic Medicine* 1986; **48**(3-4): 283-5.
20. Infurna FJ, Mayer A. The effects of constraints and mastery on mental and physical health: Conceptual and methodological considerations. *Psychol Aging* 2015; **30**(2): 432-48.
21. Weden MM, Shih RA, Kabeto MU, Langa KM. Secular Trends in Dementia and Cognitive Impairment of U.S. Rural and Urban Older Adults. *American Journal of Preventive Medicine* 2018; **54**(2): 164-72.
22. Hale JM, Schneider DC, Mehta NK, Myrskylä M. Cognitive impairment in the U.S.: Lifetime risk, age at onset, and years impaired. *SSM - Population Health* 2020; **11**: 100577.
23. Williams BD, Pendleton N, Chandola T. Cognitively stimulating activities and risk of probable dementia or cognitive impairment in the English Longitudinal Study of Ageing. *SSM - Population Health* 2020; **12**: 100656.
24. Davies HR, Cadar D, Herbert A, Orrell M, Steptoe A. Hearing Impairment and Incident Dementia: Findings from the English Longitudinal Study of Ageing. *J Am Geriatr Soc* 2017; **65**(9): 2074-81.
25. Discacciati A, Bellavia A, Lee JJ, Mazumdar M, Valeri L. Med4way: a Stata command to investigate mediating and interactive mechanisms using the four-way effect decomposition. *International Journal of Epidemiology* 2019; **48**(1): 15-20.
26. Åhlin JK, Halonen JI, Madsen IEH, Rugulies R, Sørensen JK, Magnusson Hanson LL. Interrelationships between job demands, low back pain and depression: A four-way decomposition analysis of direct and indirect effects of job demands through mediation and/or interaction. *Journal of Affective Disorders* 2021; **282**: 219-26.
27. Karlsson IK, Zhan Y, Wang Y, et al. Adiposity and the risk of dementia: mediating effects from inflammation and lipid levels. *European Journal of Epidemiology* 2022.
28. Burgos Ochoa L, Rijnhart JJM, Penninx BW, Wardenaar KJ, Twisk JWR, Heymans MW. Performance of methods to conduct mediation analysis with time-to-event outcomes. *Statistica Neerlandica* 2020; **74**(1): 72-91.
29. VanderWeele TJ. Mediation Analysis: A Practitioner's Guide. *Annual Review of Public Health* 2016; **37**(1): 17-32.
30. Mansournia MA, Altman DG. Inverse probability weighting. *BMJ* 2016; **352**: i189.
31. Chesnaye NC, Stel VS, Tripepi G, et al. An introduction to inverse probability of treatment weighting in observational research. *Clinical Kidney Journal* 2022; **15**(1): 14-20.
32. Baron RM, Kenny DA. The moderator–mediator variable distinction in social psychological research: Conceptual, strategic, and statistical considerations. *Journal of Personality and Social Psychology* 1986; **51**(6): 1173-82.
